# A combination of the percentages of IFN-γ^+^CD4^+^T cells and granzyme B^+^CD19^+^B cells is associated with acute hepatic rejection: a case control study

**DOI:** 10.1186/s12967-021-02855-w

**Published:** 2021-05-01

**Authors:** Ji-Qiao Zhu, Jing Wang, Xian-Liang Li, Wen-Li Xu, Shao-cheng Lv, Xin Zhao, Ren Lang, Qiang He

**Affiliations:** Department of Hepatobiliary and Pancreaticosplenic Surgery, Medical Research Center, Beijing Organ Transplant Center, Beijing Chaoyang Hospital, Capital Medical University, No. 8 Gongtinan Road, Chaoyang District, Beijing, 100020 China

**Keywords:** Acute rejection, Liver transplantation, B lymphocyte subsets, T lymphocyte subsets

## Abstract

**Background:**

T cells and B cells play a key role in alloimmune responses. We aimed to characterize the shift of T cell subsets and B cell subsets during acute hepatic rejection, and further determine whether they could serve as a prognostic marker.

**Methods:**

Blood samples together with the clinical data from liver transplant recipients with and without acute hepatic rejection were collected and analyzed as well as from a validation cohort.

**Results:**

Upon activation the expression of TGF-β and granzyme B in CD19^+^B cells, and the expression of IL-2 and IFN-γ in CD4^+^T cells were higher in acute hepatic rejection. However, only the frequencies of granzyme B^+^CD19^+^B cells and IFN-γ^+^CD4^+^T cells correlated with liver function in addition to with each other. A combination of the two cell subsets as a novel marker could classify rejection versus non-rejection (area under the curve 0.811, p = 0.001) with the cut-off value of 62.93%, which was more sensitive for worse histological changes (p = 0.027). Moreover, the occurrence rate of acute rejection was higher in the group with the novel marker > 62.93% (p = 0.000). The role of the novel marker was further confirmed in a validation cohort, which was identified to be the only significant independent risk factor for acute rejection (odds ratio: 0.923; 95% CI confidence interval: 0.885–0.964; p = 0.000).

**Conclusions:**

A combination of the percentages of IFN-γ^+^CD4^+^T cells and granzyme B^+^CD19^+^B cells can distinguish rejection from non-rejection, which can be used as a potential prognostic marker for acute rejection in liver transplant recipients.

**Supplementary Information:**

The online version contains supplementary material available at 10.1186/s12967-021-02855-w.

## Introduction

Liver transplantation is a major therapeutic approach in patients with end-stage liver diseases [1]. Following transplantation acute rejection remains a major challenge despite the development of immunosuppressive drugs [2]. Although abnormalities in liver function may raise concerns about acute rejection, invasive liver biopsies are still the gold standard for definitive diagnoses [3]. Therefore, a potential biomarker with minimal invasion for the diagnosis of acute rejection after liver transplantation has been required. Actually, acute rejection is realized via the immune cells, thus, blood-derived biomarkers are under intensive research.

By means of producing different cytokines and exerting different biological functions, CD4^+^T cells are thought to play a crucial role during the rejection process [4, 5]. Tang et al. reported IL-2 and IFN-γ could be produced by memory CD4^+^T-helper cells to mediate vigorous allograft rejection [6]; Sawitzki B et al. found IFN-γ secreted by CD4^+^T cells was responsible for transplantation tolerance in a skin transplantation model [7]. In another study transplantation of older organs triggered more potent alloimmune responses via proinflammatory cytokine production of IL-17 [8]. Immature dendritic cells-derived exosomes improved the percent of survival and suppressed rejection associated cytokines IFN-γ, IL-2, IL-17 production [9]. Hence, CD4^+^T cells can promote the rejection process.

In the meanwhile, B cells were once considered to act as antigen-presenting cells and provide co-stimulatory signals for T cell activation in addition to antibody production. Recent studies have demonstrated the production of multiple cytokines can enable B cells to regulate the immune response [10, 11]. IL-10 is a typical cytokine produced by regulatory B cells, which suppresses the proliferation and inflammatory cytokine productions of effector T cells [12]. In our previous study, Granzyme B (GrB) produced by B cells was found to maintain allospecific tolerance in patients with renal rejection [13]. Moreover, low mRNA expression of TGF-β in the biopsy could reveal early acute rejection implying an increased risk for renal graft failure [14]. Therefore, regulatory B cells are characterized by the immunosuppressive function.

However, the dynamic changes of T cell subsets and B cell subsets during acute rejection have not been well elucidated. Furthermore, there has not been a widely accepted biomarker for predicting acute rejection. Thus, we performed the study to investigate whether the T cell subsets and B cell subsets could serve as a prognostic marker in liver transplant recipients (LTR) with acute rejection.

## Materials and methods

### Study design

LTR with acute rejection and with stable liver function were enrolled in this study, who underwent a first single liver transplant or were followed up at Beijing Chaoyang Hospital. There were not any signs of postoperative complications in all cases at the time of blood sampling except acute rejection. The controls did not have an episode of acute rejection if the follow-up period was ≤ 6 months; otherwise, the period between acute rejection and sampling was > 6 months. A validation cohort was further enrolled to confirm the observations. The study was approved by the Institutional Review Board of Beijing Chaoyang Hospital (No. 2016-2-19-38) in accordance with the Helsinki declaration of 1975, as revised in 1983. Written informed consent was obtained from all participants.

### Immunosuppressive management

Immunosuppressive therapy contained induction with basiliximab (20 mg on day 0 and day 4) and maintenance on calcineurin inhibitors. Acute rejection was diagnosed using clinical and laboratory parameters and graft biopsy assessed according to the Banff schema [15]. LTR with acute rejection were treated by adding the dose of immunosuppressants or with a round of steroids (Methylprednisolone given 500 mg on day 1, 240 mg on day 2, then daily reduced by 40 mg till the 8th day, finally changed to prednisolone 20 mg/day for ~ 1 month).

### Cell culture

Lymphocyte subset analysis was performed at the time of biopsy before the therapeutic intervention. Peripheral blood mononuclear cells (PBMC) were isolated by ficoll density gradient centrifugation and suspended (1 × 10^6^/ml) in RPMI-1640 medium supplemented with 10% heat inactivated fetal calf serum, 100 U/ml penicillin, 100 μg/ml Streptomycin (Invitrogen). For cytokines produced by B cells, PBMC were stimulated with IgG + IgM (5.4 μg/ml) in the presence of IL-21 (50 ng/ml), seeded in a 24-well flat-bottom plate, and incubated for 20 h at 37 °C, 5% CO_2_. Brefeldin A (1 μg/ml) was added for the last 4 h of incubation. For cytokines produced by T cells, PBMC (1 × 10^6^/ml) were stimulated with cell stimulation cocktail (eBioscience) and seeded in a 15-ml tube and incubated for 4 h at 37 °C, 5% CO_2_.

### Antibodies and flow cytometric measurement

Anti-human mAbs included PE-Cy7-CD3 (Biolegend), PerCP-Cy5.5-CD8 (Biolegend), APC-IL-17 (eBioscience), PE-IFN-γ (Biolegend), APC-IL-2 (Biolegend), PE-Cy7-CD19 (Biolegend), FITC-Zombie (Biolegend), APC-fire750-CD69 (Biolegend), PerCP-Cy5.5-TGF-β (Biolegend), APC-IL-10 (Biolegend), PE-GrB (eBioscience). After stimulation, PBMC were harvested and first stained with surface antibodies followed by fixation/permeabilization (Cytofix/Cytoperm kit, BD Biosciences) and subsequent intracellular staining, as previously described [13].

Flow cytometry was performed in NovoCyte D2060R (ACEA Biosciences Inc). NovoEXpress software (San Diego, CA, USA) was used for analysis. Flow cytometry characterization of lymphocyte subsets is presented in Additional file 1: Figure S1.

### Statistical analysis

Data analyses were carried out by using SPSS 19.0 computer software (IBM Corp., Armonk, NY, USA) and GraphPad Prism 5 software (GraphPad Software Inc., La Jolla, CA, USA). Values were expressed as mean ± standard deviation. The Kolmogorov–Smirnov test was used for the normal distribution of continuous variables. The independent samples t-test was employed for quantitative variables. The Mann–Whitney U test was selected due to non-normal distribution. The Chi-square or Fisher’s exact test was used to compare nominal variables. Receiver operating characteristic curve (ROC) analysis and comparison of the area under the curve (AUC) was performed. The cut-off value for positive parameters was further determined by optimal sensitivity and specificity on ROC curve analysis. Multivariate Cox analysis was employed to determine the predictors. Relative risk was expressed as an odds ratio (OR) with a 95% confidence interval (CI). A p-value < 0.05 was considered statistically significant.

## Results

### Characteristics of LTR

A total of 15 LTR with acute rejection and 30 paired controls with stable liver function (1:2) were enrolled in this study. They were matched by gender, age (± 3 years), primary diseases for transplantation (benign or malignant), main immunosuppressants, and follow-up periods (± 7 days). Patients were all first deceased donor allograft recipients. The characteristics of LTR are listed in Additional file 1: Table S1. Of the 15 patients, 13 had T cell-mediated rejection proven by histological evidence and two mixed T cell and antibody-mediated rejection. Six patients were treated with adding the dose of immunosuppressants alone and nine followed by a round of steroids. All patients recovered with stable liver function.

### The percentages of IFN-γ^+^CD4^+^T cells and IL-2^+^CD4^+^T cells could differentiate rejection from non-rejection upon activation

**Fig. 1:**
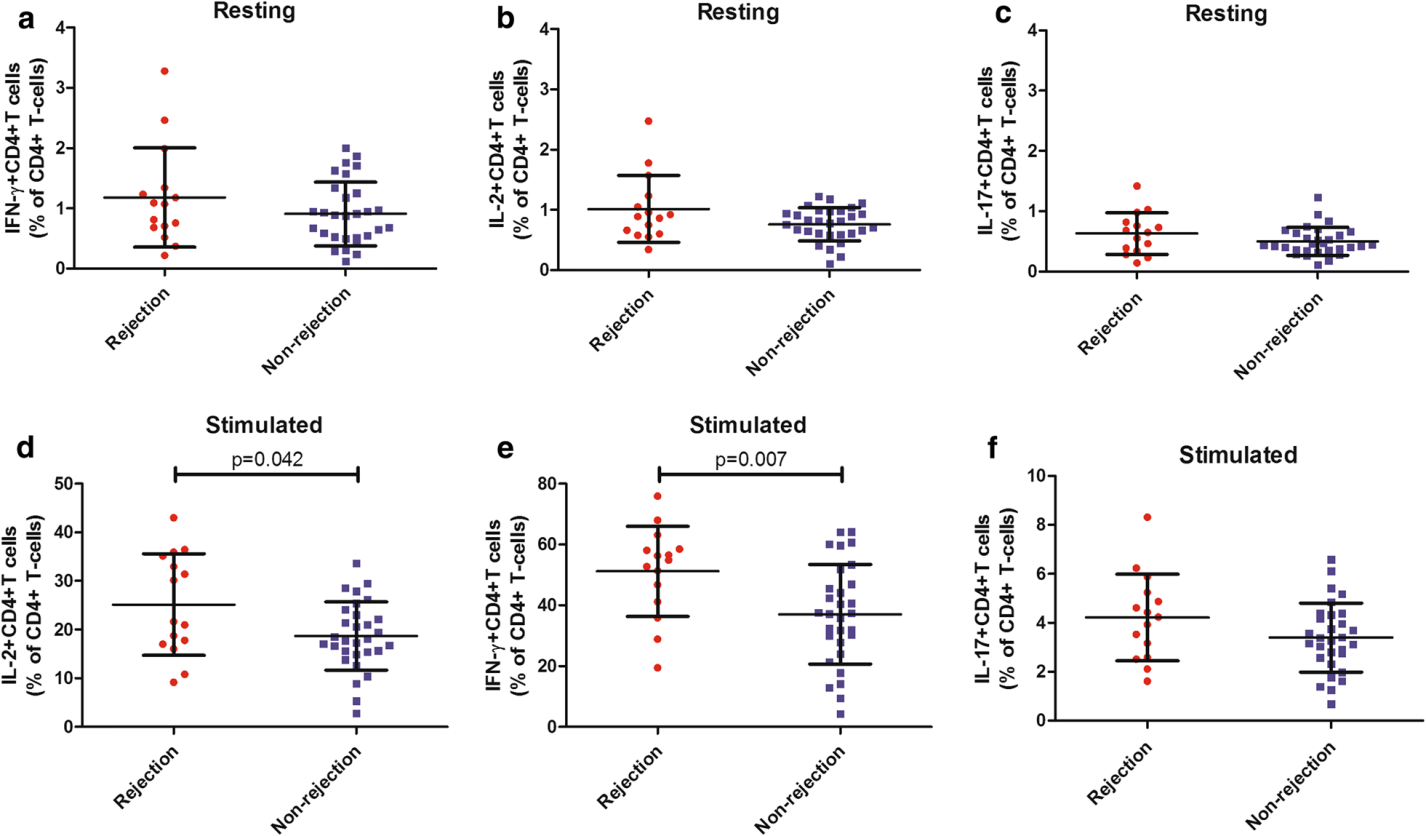
The percentages of IFN-γ^+^CD4^+^T cells and IL-2^+^CD4^+^T cells could differentiate rejection from non-rejection upon activation. Comparison of the production of IFN-γ, IL-2 and IL-17 in resting CD4^+^T cells (**a**–**c**) and in CD4^+^T cells stimulated with cell stimulation cocktail (**d**–**f**) between liver transplant recipients with and without acute rejection. Bars represent mean and standard deviation

CD4^+^T cells have been reported to play an important role in allograft rejection [16, 17] while the dynamic changes of T lymphocyte subsets have not been well investigated in LTR. Therefore, we wanted to check the shift of circulating T cells. After analysis, we found the production of IFN-γ, IL-2, and IL-17 in resting CD4^+^T cells increased in LTR with rejection but did not reach statistical significance between the two groups (P > 0.05, Fig. 1a–c). When activated with stimulation cocktail, CD4^+^Tcell derived IL-2 production (p = 0.042, Fig. 1d) and IFN-γproduction (p = 0.007, Fig. 1e) rose significantly in LTR with rejection; meanwhile, the percentages of IL-17^+^ CD4^+^T cells remained similar (P > 0.05, Fig. 1f) between the two groups (Additional file 1: Table S2).

### The percentages of GrB^+^CD19^+^B cells and TGF-β^+^CD19^+^B cells could differentiate rejection from non-rejection upon activation

**Fig. 2:**
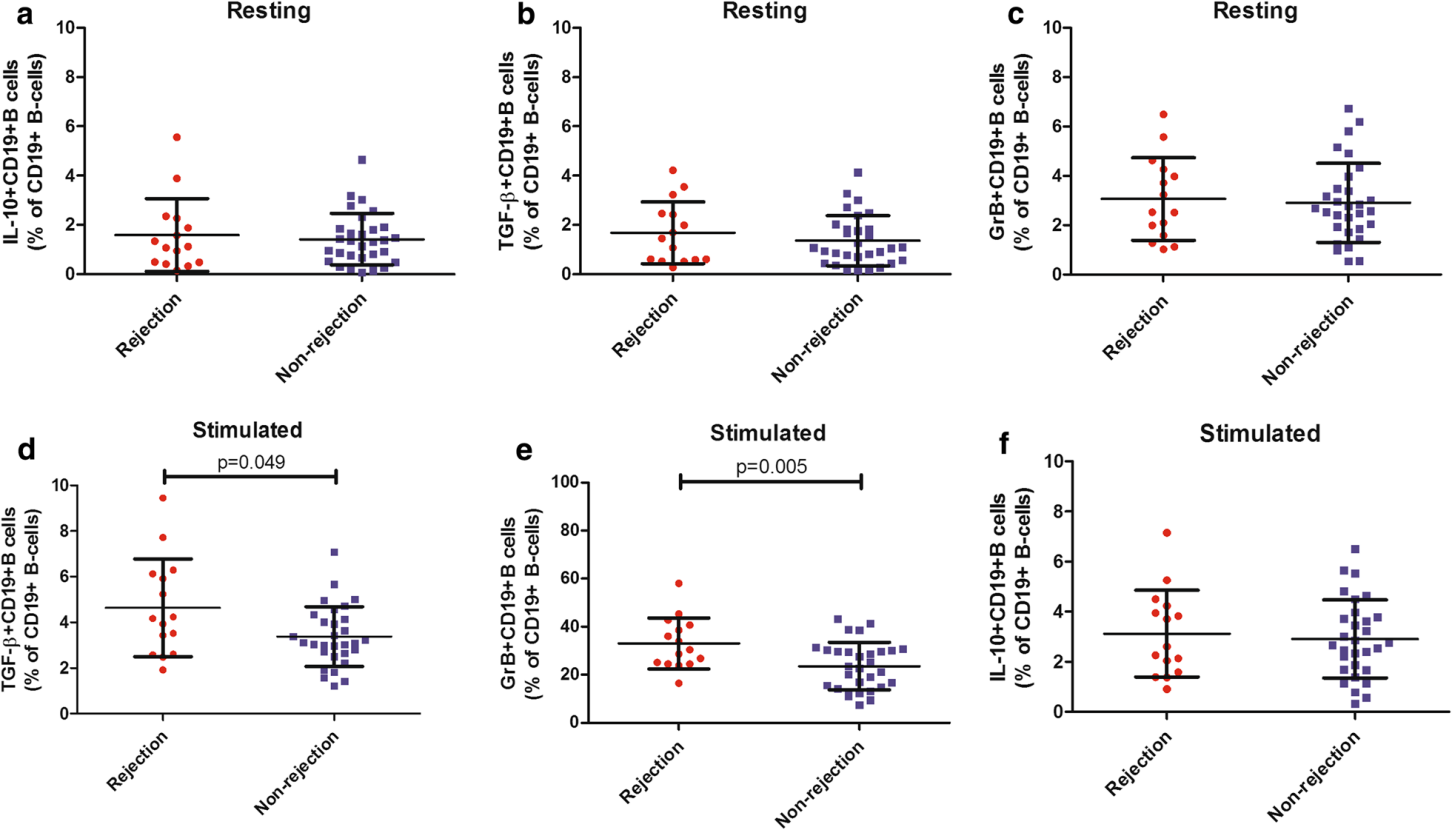
The percentages of GrB^+^CD19^+^B cells and TGF-β^+^CD19^+^B cells could differentiate rejection from non-rejection upon activation. Comparison of the production of IL-10, TGF-β and GrB in resting CD19^+^B cells (**a**–**c**) and in CD19^+^B cells stimulated with IgG + IgM in the presence of IL-21 (**d**–**f**) between liver transplant recipients with and without acute rejection. Bars represent mean and standard deviation. GrB, granzyme B

We previously showed that the frequency of GrB^+^CD19^+^B cells increased in patients with renal rejection [13]. In this study, we extended our research to determine how various B cell subsets would shift in LTR with acute rejection. First, we detected the expression of IL-10, TGF-β, and GrB in circulating B cells. The results showed the production of IL-10, TGF-β and GrB in resting CD19^+^B cells was similar between the two groups, respectively (P > 0.05, Fig. 2a–c). Next, B cells were stimulated with anti-IgG/IgM in the presence of IL-21. We observed an obvious increase in the percentages of TGF-β^+^CD19^+^B cells (p = 0.049, Fig. 2d) and GrB^+^CD19^+^B cells (p = 0.005, Fig. 2e) from LTR with acute rejection while the percentage of IL-10^+^CD19^+^B cells (P > 0.05, Fig. 2f) was comparable upon stimulation between two groups (Additional file 1: Table S3).

### The percentages of GrB^+^CD19^+^B cells and IFN-γ^+^CD4^+^Tcells correlate with liver function

**Fig. 3:**
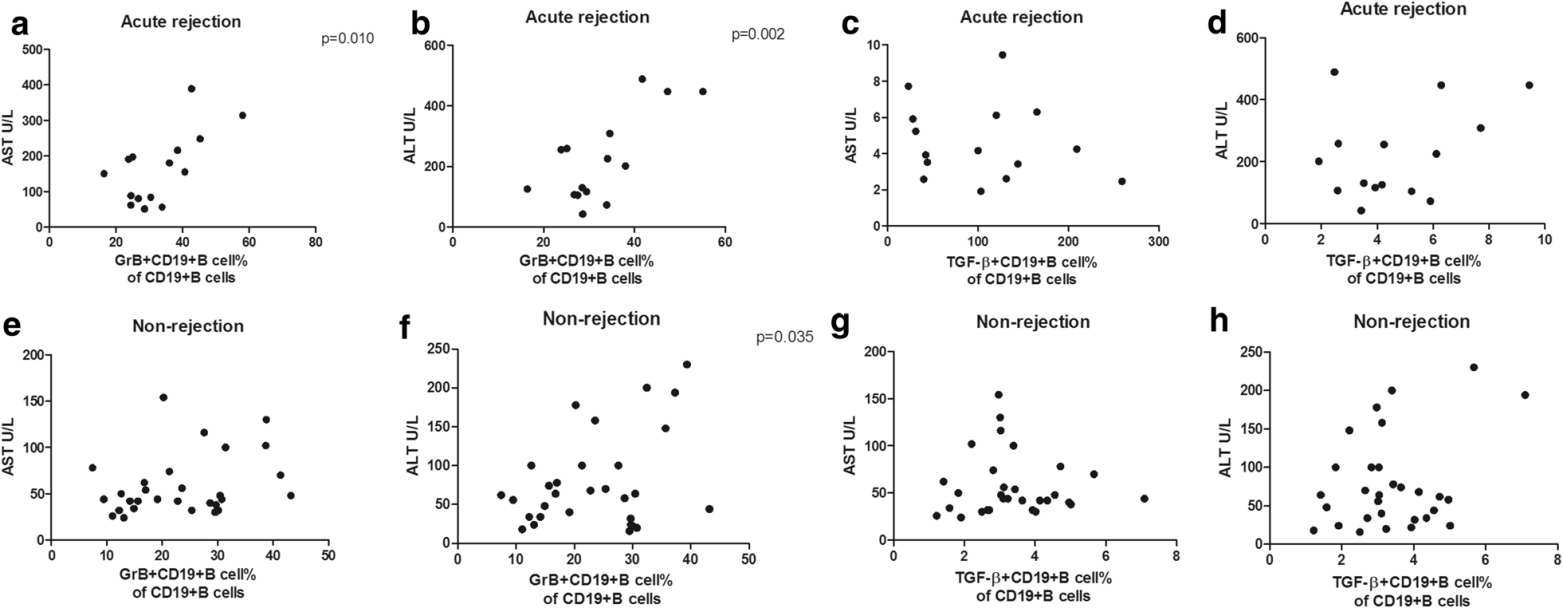
The percentages of GrB^+^CD19^+^B cells correlate with liver function upon activation. Correlations of AST and ALT, and the percentages of GrB^+^CD19^+^B cells in liver transplant recipients with (**a**, **b**) and without (**e**, **f**) acute rejection, respectively. Correlations of AST and ALT, and the percentages of TGF-β^+^CD19^+^B cells in liver transplant recipients with (**c**, **d**) and without (**g**, **h**) acute rejection, respectively. Bars represent mean and standard deviation. *GrB* granzyme B, *AST* aspartate transaminase, *ALT* alanine amiotransferase

**Fig. 4:**
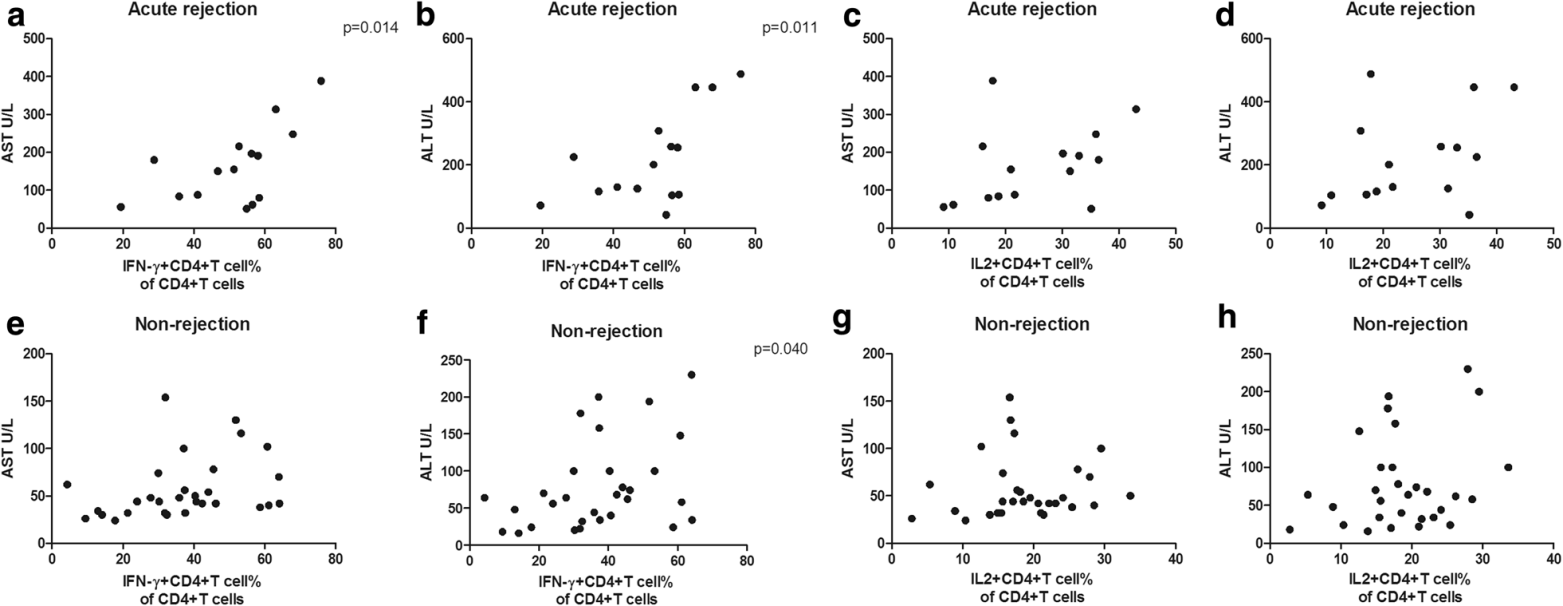
The percentages of IFN-γ^+^CD4^+^T cells correlate with liver function upon activation. Correlations of AST and ALT, and the percentages of IFN-γ^+^CD4^+^T cells in liver transplant recipients with (**a**, **b**) and without (**e**, **f**) acute rejection, respectively. Correlations of AST and ALT, and the percentages of IL-2^+^CD4^+^T cells in liver transplant recipients with (**c**, **d**) and without (**g**, **h**) acute rejection, respectively. Bars represent mean and standard deviation. *AST* aspartate transaminase, *ALT* alanine amiotransferase

Since activated cell subsets could differentiate rejection from non-rejection, we wanted to know whether a sharp increase in the subset percentages was in close relation to liver function. First, clinical data from LTR with and without rejection were collected and compared (Additional file 1: Table S4). LTR with acute rejection had higher levels of aspartate transaminase (AST, p = 0.001, Additional file 1: Figure S2A) and alanine amiotransferase (ALT, p = 0.002, Additional file 1: Figure S2B). Surprisingly, levels of TBIL were comparable (Figure S2C). We attributed the TBIL change to the diagnosis of acute rejection at an early stage as the rise of transaminases was more rapid. Notably, tacrolimus levels were also similar between the two groups (Additional file 1: Figure S2D), implying the similar immunosuppressive therapy of the two groups. Then, we performed the correlation analysis between activated cell subsets and the transaminases. We found the percentages of GrB^+^CD19^+^B cells correlated positively with AST (p = 0.010, Fig. 3a) and ALT (p = 0.002, Fig. 3b) in LTR with acute rejection, and with ALT (p = 0.035, Fig. 3f) in LTR without rejection. Such a correlation was not found between AST and ALT, and the percentages of TGF-β^+^CD19^+^B cells either in LTR with rejection (Fig. 3c, d) or in LTR without rejection (Fig. 3g, h) as well as the percentages of GrB^+^CD19^+^B cells and AST in LTR without rejection (Fig. 3e). Similarly, the percentages of IFN-γ^+^CD4^+^T cells correlated positively with AST (p = 0.014, Fig. 4a) and ALT (p = 0.011, Fig. 4b) in LTR with acute rejection and with ALT (p = 0.040, Fig. 4f) in LTR without rejection. In contrast, the percentages of IL-2^+^CD4^+^T cells did not correlate with AST and ALT either in LTR with rejection (Fig. 4c, d) or in LTR without rejection (Fig. 4g, h). Neither did the percentages of IFN-γ^+^CD4^+^T cells correlate with AST in LTR without rejection (Fig. 4e).

### A combination of the percentages of IFN-γ^+^CD4^+^T cells and GrB^+^CD19^+^B cells as a novel marker can identify acute rejection

**Fig. 5:**
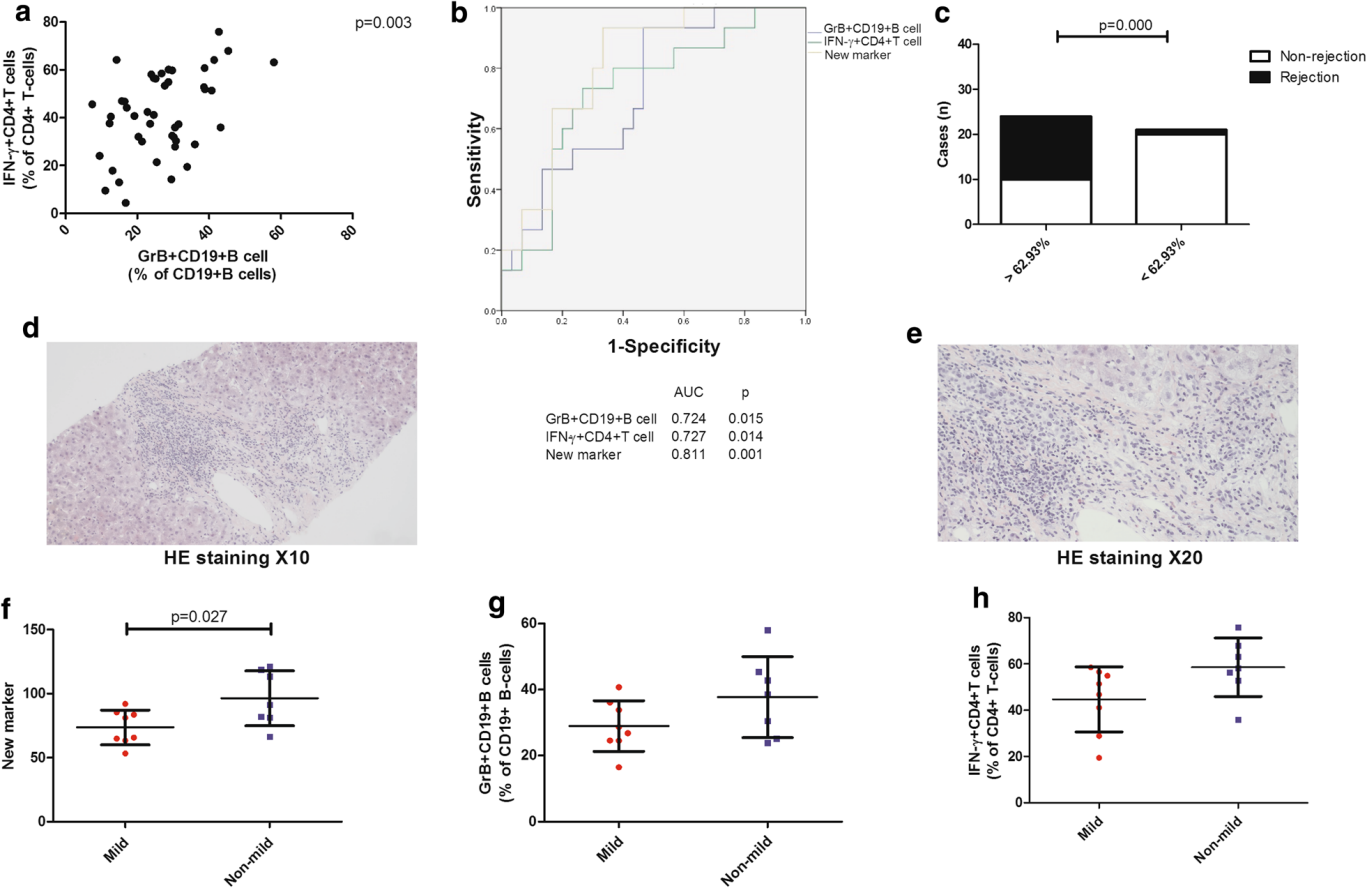
A combination of the percentages of IFN-γ^+^CD4^+^T cells and GrB^+^CD19^+^B cells as a novel marker can identify acute rejection. Correlations of the percentages of IFN-γ^+^CD4^+^T cells and the percentages of GrB^+^CD19^+^B cells (**a**); ROC curve analysis of percentages of IFN-γ^+^CD4^+^T cells and GrB^+^CD19^+^B cells and a combination of both as the new marker (**b**); The occurrence rate of acute rejection between liver transplant recipients with the novel marker > 62.93% and < 62.93% (**c**); Representative images of hematoxylin–eosin staining from liver transplant recipients with acute cellular rejection (**d**, **e**); Analysis of the percentages of IFN-γ^+^CD4^+^T cells and GrB^+^CD19^+^B cells and the novel marker between liver transplant recipients with mild and non-mild inflammation and damage (**f**, **h**). Bars represent mean and standard deviation. *GrB* granzyme B, *ROC* receiver operating characteristic, *AUC* area under the curve

From the above analysis, the percentages of activated IFN-γ^+^CD4^+^T cells and activated GrB^+^CD19^+^B cells were determined to have a strong association with acute rejection. Besides, we also observed a positive correlation between the percentages of activated IFN-γ^+^CD4^+^T cells and the percentages of activated GrB^+^CD19^+^B cells after further analysis (Fig. 5a). Based on their relationship we proposed a novel marker of a combination of the two cell subsets and tested its efficacy.

First, we performed the analysis of AUC-ROC for the percentages of activated IFN-γ^+^CD4^+^T cells and the percentages of activated GrB^+^CD19^+^B cells and the new marker (Fig. 5b), which revealed the latter strongly classified rejection versus non-rejection (AUC 0.811, p = 0.001) with the cut-off value of 62.93%. The corresponding sensitivity and specificity were 93.3% and 66.7%, respectively. Then, the LTR were regrouped according to the cut-off value. We found the occurrence rate of acute rejection was higher in the group with the new marker > 62.93% (p = 0.000; Fig. 5c). Finally, the association between three parameters and the severity of the Banff histological grades (mild group and non-mild group) was analyzed (Fig. 5d, e). Of note, LTR with mild inflammation and damage had low values of the new marker (p = 0.027, Fig. 5f). While the percentages of activated IFN-γ^+^CD4^+^T cells and activated GrB^+^CD19^+^B cells failed to identify the LTR (Table S5, Fig. 5g, h). Thus, the novel marker not only identified LTR with acute rejection but also was more sensitive for the worse acute histological changes.

### The novel marker can predict acute rejection in a validation cohort

In an attempt to confirm the ability of the novel marker to identify LTR with rejection, we performed an independent study. In this validation cohort, a total of 101 LTR were enrolled, who accepted liver transplantation or were followed up at our center within one year after surgery. Blood samples were obtained to analyze the lymphocyte subsets. These patients were followed up for at least one year to observe the occurrence of acute rejection as most acute rejections occur within the first year following liver transplantation [18–20]. 23 LTR had a rejection episode during the follow-up period and were treated by adding the dose of immunosuppressants or with a round of steroids.

**Fig. 6:**
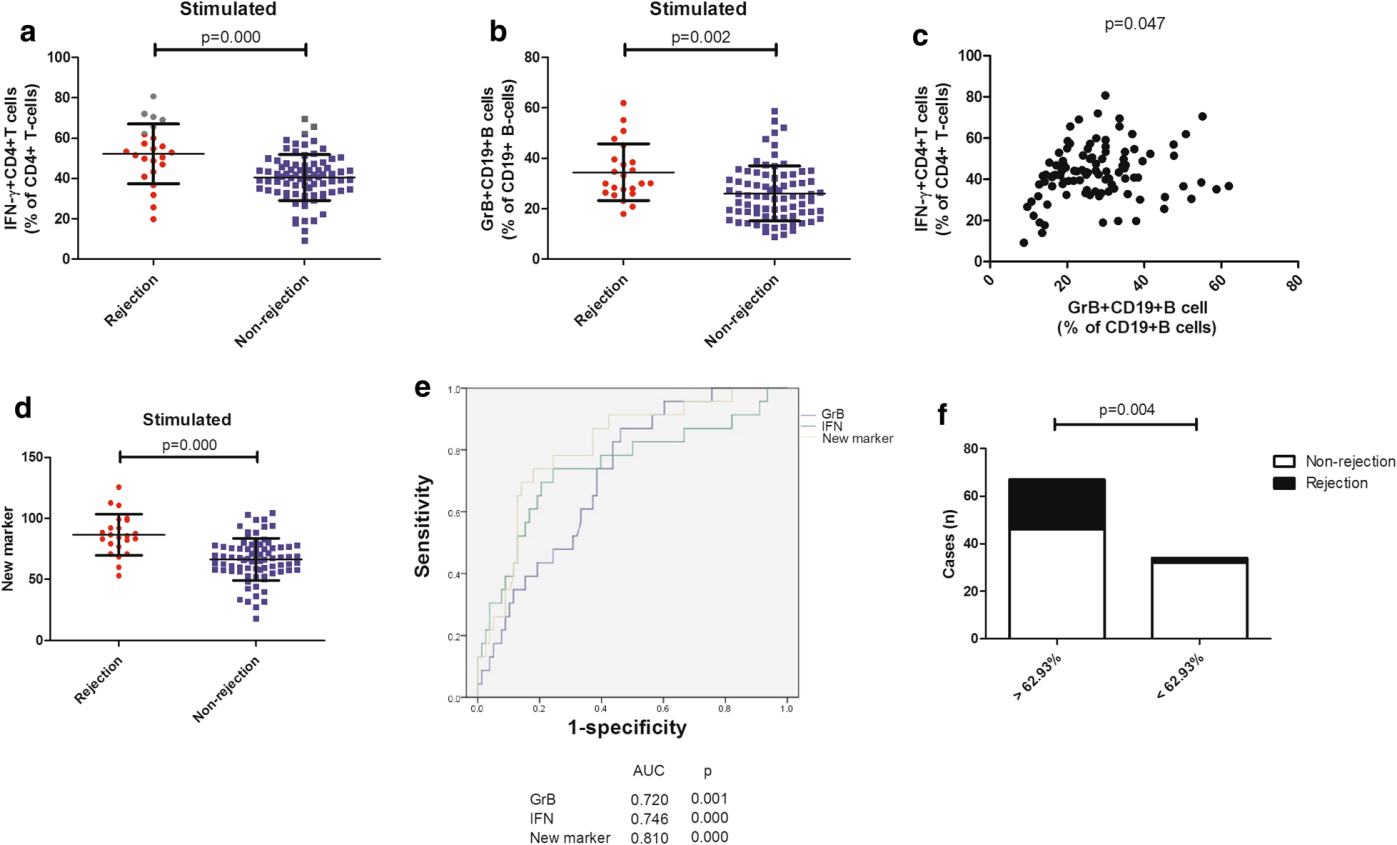
The novel marker can predict acute rejection in a validation cohort. Comparison of the percentages of IFN-γ^+^CD4^+^T cells and GrB^+^CD19^+^B cells between liver transplant recipients with and without acute rejection (**a**, **b**); Correlations of the percentages of IFN-γ^+^CD4^+^T cells and the percentages of GrB^+^CD19^+^B cells (**c**); Comparison of the novel marker between liver transplant recipients with and without acute rejection (**d**); ROC curve analysis of the percentages of IFN-γ^+^CD4^+^T cells and GrB^+^CD19^+^B cells and the novel marker (**e**); The occurrence rate of acute rejection between liver transplant recipients with new marker > 62.93% and < 62.93% (**f**). Bars represent mean and standard deviation. *GrB* granzyme B, *ROC* receiver operating characteristic, *AUC* area under the curve

In this validation cohort, LTR with acute rejection had the higher percentages of activated IFN-γ^+^CD4^+^T cells (p = 0.000, Fig. 6a) and activated GrB^+^CD19^+^B cells (p = 0.002, Fig. 6b). Again, the percentages of activated IFN-γ^+^CD4^+^T cells and the percentages of activated GrB^+^CD19^+^B cells correlated with each other (p = 0.047, Fig. 6c). We considered that might explain the effect of the new marker, which showed higher values in LTR with rejection (p = 0.000, Fig. 6d, Additional file 1: Table S6). Subsequent ROC analysis further confirmed that the new marker was a strong predictor of acute rejection with an AUC of 0.810 (Fig. 6e). When the LTR were regrouped at the cut-off value of 62.93%, the occurrence rate of acute rejection remained higher in the group with the new marker > 62.93% (p = 0.004; Fig. 6f). Finally, we asked if the new marker could independently predict acute rejection. On univariate analysis age (p = 0.009), CMV infection (0.049), postoperative lymphocyte percentage (p = 0.020), and the new marker (p = 0.000) were identified to be positive between LTR with and without rejection. Comparison of other parameters did not reach significance (p > 0.05). Using multiple logistic regression analysis, the new marker (OR: 0.923; 95% CI: 0.885–0.964; p = 0.000) was the only significant independent risk factor (Table 1).

**Table 1:** Risk factors for acute rejection in liver transplant recipients

Parameters	Univariate analysis			Multivariate analysis		
	Rejection (n = 23)	Non-rejection (n = 78)	P	OR	CI	P
Sex (male)	22	71	0.777			
Age	49.09 ± 9.91	54.19 ± 7.40	0.009	1.073	0.997–1.154	0.060
Primary disease			0.821			
Hepatitis related cirrhosis	13	42				
Hepacellular carcinoma	10	36				
Preoperative creatinine (µmol/l)	65.43 ± 17.85	73.84 ± 28.69	0.364			
Preoperative bilirubin (µmol/l)	52.80 ± 71.58	72.52 ± 142.72	0.827			
Preoperative INR	1.20 ± 0.27	1.30 ± 0.30	0.160			
Preoperative lymphocyte count (10^9/l)	1.26 ± 0.80	1.09 ± 0.71	0.331			
Preoperative lymphocyte percentage	25.49 ± 13.64	24.11 ± 11.94	0.639			
Operating time (min)	401.22 ± 282.80	312.68 ± 288.11	0.176			
Warm ischemia time (min)	2.48 ± 0.85	2.63 ± 0.69	0.521			
Cold storage time (h)	7.35 ± 1.03	7.62 ± 1.06	0.307			
Bleeding (> 800 ml)	12	39	0.855			
Transfusion (> 800 ml)	5	31	0.113			
Anhepatic phase (min)	127.74 ± 26.45	118.99 ± 35.11	0.272			
Immunosuppressants			0.484			
Tacrolimus-based	19	59				
Cyclosporin A-based	4	19				
Tacrolimus level (ng/ml)	6.04 ± 4.99	7.35 ± 5.63	0.365			
CMV	9	15	0.049	2.355	0.700–7.930	0.167
Postoperative lymphocyte count (10^9/l)	1.41 ± 1.04	1.19 ± 0.72	0.252			
Postoperative lymphocyte percentage	23.88 ± 8.23	19.10 ± 8.56	0.020	0.962	0.902–1.027	0.246
New marker	86.62 ± 16.76	66.48 ± 17.33	0.000	0.923	0.885–0.964	0.000

*INR* international normalized ratio, *CMV* cytomegalovirus, *OR* odds ratio, *CI* 95% confidence interval

## Discussion

In this study, we found that a combination of percentages of activated IFN-γ^+^CD4^+^T cells and activated GrB^+^CD19^+^B cells can distinguish rejection from non-rejection, which can be used as a novel biomarker for diagnosis of acute rejection in LTR.

IL-17 is a pro-inflammatory cytokine implicated in the pathogenesis of lung and renal transplantation as reduced IL-17 production was associated with attenuation of acute rejection [21, 22]. Similar results were reported in liver transplantation [23, 24]. In contrast, we found high IL-17 production in LTR with rejection upon activation, which failed to reach significance. Notably, we detected the percentages of IL-17^+^CD4^+^T cells rather than serum levels of IL-17 in this study. In addition, LTR with and without rejection were all found to have higher levels of IL-17 than healthy controls during the postoperative period [25], which might minimize the difference. IL-2 and IFN-γ are typically secreted by activated CD4^+^T cells, specifically T-helper 1 cells, which play an important role in acute rejection. Our findings are in line with reported studies stressing the function of IL-2 and IFN-γ during the rejection process [26–28]. They also showed LTR with improved liver function had low levels of IL-2 and IFN-γ. However, the correlation analysis has not been investigated among their studies. We first demonstrated the percentages of activated IFN-γ^+^CD4^+^T cells instead of activated IL-2^+^CD4^+^T cells was found to positively correlate with transaminases in both the rejection group and the non-rejection group. A possible explanation for the difference between the two cytokines might be their main functions. IL-2 promotes the proliferation and differentiation of lymphocytes [29, 30]; while IFN-γ can induce hepatocyte apoptosis or inhibit hepatocyte cell cycle progression [31].

Recent studies have revealed that B cells can regulate the immune response via immunosuppressive cytokine secretion in addition to producing antibodies and presenting antigens to T cells [32]. Due to its immunosuppressive nature, regulatory B cells have been repeatedly reported to be involved in transplantation tolerance [33, 34]. In contrast, its regulation in acute rejection following transplantation is poorly understood. In our study, we found LTR with acute rejection had higher percentages of TGF-β^+^CD19 + B cells and GrB^+^CD19^+^B cells, which might affect as feedback to main tolerance [35]. Surprisingly, the percentage of IL-10^+^CD19^+^B cells was similar to controls. Although TGF-β, GrB, and IL-10 are all produced by regulatory B cells they belong to distinct cell subsets (data not shown). The most efficient way for IL-10 expression is to stimulate B cells through TLR signaling activation while maximal TGF-β and GrB production is induced through BCR [13, 36–38]. When we further analyzed the correlations between cytokines and liver function, only the percentages of GrB^+^CD19^+^B cells showed an association with transaminases. TGF-β can regulate both cell differentiation and cell survival while GrB can strongly induce T cell apoptosis in addition to suppressing CD4^+^T cell proliferation [39, 40]. Thus, higher values of transaminases call for higher production of GrB during the rejection process.

Of note, the percentages of GrB^+^CD19^+^B cells correlated positively with the percentages of IFN-γ^+^CD4^+^T cells. As they are pro-inflammatory cytokine and anti-inflammatory cytokine, respectively, these two kinds of cytokines might work as a loop as discussed above. Therefore, we proposed a combination of the cell subsets as a new marker based on their relationship. We also showed the new marker had strong predictive capacity after analysis of AUC. When regrouped by the cut-off value, 93.3% LTR exhibiting rejection had a high value and 6.7% a low value. Furthermore, LTR with a high value of the novel marker also had worse histological scores, confirming its association with the severity of rejection. Taken together, our data suggest that the new marker is more sensitive for the risk of acute rejection.

The function of the new marker was further validated in an independent cohort. The results from the validation cohort showed the new marker could identify LTR with a very low risk for rejection from LTR who were at substantial risk for developing acute rejection. We found the new marker was identified to be the only independent predictor for rejection, which outperformed the other parameters. Several biomarkers have been shown to predict clinical acute rejection, including primary biliary cirrhosis, younger age, hepatitis C [41], IL-10-1082 polymorphism [42], de novo donor-specific antibody [20], and cytokine promoter polymorphisms [43]. However, all these risk factors need prospective validation. Notably, tacrolimus levels were comparable between LTR with and without acute rejection in both cohorts. Since the LTR received similar immunosuppressive therapy, then, rapid recovery of lymphocyte subsets might account for this phenomenon.

In conclusion, a combination of the percentages of IFN-γ^+^CD4^+^T cells and granzyme B^+^CD19^+^B cells can distinguish rejection from non-rejection, which can be used as a potential prognostic marker for acute rejection in liver transplant recipients.

## Supplementary Information

**Additional file 1**: **Table S1**. Characteristics of liver transplantrecipients with and without acute rejection. **Table S2**. Comparison of the percentages of T cell subsets between liver transplant recipients with and without rejection. **Table S3**. Comparison of the percentages of B cell subsets between liver transplant recipients with and without rejection. **Table S4.** Analysis of liver function and tacrolimus levels between liver transplant recipients with and without rejection. **Table S5**. Comparison of the Banff grades in the rejection group with the percentages of GrB+CD19+B cell and IFN-γ+CD4+T cell and the new marker. **Table S6.** Comparison of the percentages of GrB+CD19+B cell and IFN-γ+CD4+T cell and the new marker in a validation cohort. **Figure S1.** Flow cytometry characterization of T cell subsets and B cell subsets. **Figure S2.** AST and ALT deteriorate in liver transplant recipients with acute rejection. Comparison of levels of AST (A), ALT (B), TBIL (C) and FK506 (D) between liver transplant recipients with and without acute rejection. Bars represent mean and standard deviation. AST, aspartate transaminase; ALT, alanine amiotransferase; TBIL, total bilirubin.

## Data Availability

All data generated or analyzed during this study are included in this published article and its supplementary information files.

## Abbreviations

GrB: Granzyme B
PBMC: Peripheral blood mononuclear cells
ROC: Receiver operating characteristic
AUC: Area under the curve
OR: Odds ratio
CI: Confidence interval
AST: Aspartate transaminase
ALT: Alanine aminotransferase
TBIL: Total bilirubin
