# Supplementary material for: A combination of the percentages of IFN-γ+CD4+T cells and granzyme B+CD19+B cells is associated with acute hepatic rejection: a case control study
Source: J Transl Med. 2021 May 1;19:187. doi: 10.1186/s12967-021-02855-w (PMC8088570; doi:10.1186/s12967-021-02855-w)
Supplement: Supplementary file 1 — Additional file 1: Table S1. Characteristics of liver transplantrecipients with and without acute rejection. Table S2. Comparison of the percentages of T cell subsets between liver transplant recipients with and without rejection. Table S3. Comparison of the percentages of B cell subsets between liver transplant recipients with and without rejection. Table S4. Analysis of liver function and tacrolimus levels between liver transplant recipients with and without rejection. Table S5. Comparison of the Banff grades in the rejection group with the percentages of GrB+CD19+B cell and IFN-γ+CD4+T cell and the new marker. Table S6. Comparison of the percentages of GrB+CD19+B cell and IFN-γ+CD4+T cell and the new marker in a validation cohort. Figure S1. Flow cytometry characterization of T cell subsets and B cell subsets. Figure S2. AST and ALT deteriorate in liver transplant recipients with acute rejection. Comparison of levels of AST (A), ALT (B), TBIL (C) and FK506 (D) between liver transplant recipients with and without acute rejection. Bars represent mean and standard deviation. AST, aspartate transaminase; ALT, alanine amiotransferase; TBIL, total bilirubin. [file 12967_2021_2855_MOESM1_ESM.doc]

Additional file 1

Table S1. Characteristics of liver transplantrecipients with and without acute rejection

| Parameters | Rejection (n=15) | Non-rejection (n=30) | p |
| --- | --- | --- | --- |
| Age | 49.20±10.54 | 50.97±10.85 | 0.606 |
| Sex (male) | 13 | 26 | 1.000 |
| Primary disease |  |  | 1.000 |
| Cirrhosis | 8 | 16 |  |
| Cancer | 7 | 14 |  |
| Induction (basiliximab) | 15 | 30 | 1.000 |
| Maintenance |  |  | 1.000 |
| Tacrolimus | 14 | 28 | 1.000 |
| Cyclosporin A | 1 | 2 |  |
| Follow-up period | 53.60±27.95 | 56.60±28.97 | 0.742 |

Table S2. Comparison of the percentages of T cell subsets between liver transplant recipients with and without rejection

| Percentages (%) | Rejection (n=15) | Non-rejection (n=30) | P |
| --- | --- | --- | --- |
| ^a^IFN-γ^+^CD4^+^T cell | 1.18±0.92 | 0.91±0.53 | 0.212 |
| ^a^IL-2^+^CD4^+^T cell | 1.01±0.55 | 0.76±0.28 | 0.114 |
| ^a^IL-17^+^CD4^+^T cell | 0.63±0.35 | 0.50±0.24 | 0.141 |
| ^b^IFN-γ^+^CD4^+^T cell | 51.17±14.80 | 37.05±16.42 | 0.007 |
| ^b^IL-2^+^CD4^+^T cell | 25.15±10.44 | 18.70±6.99 | 0.042 |
| ^b^IL-17^+^CD4^+^T cell | 4.22±1.77 | 3.39±1.41 | 0.097 |

a, resting T cells; b, activated T cells

Table S3. Comparison of the percentages of B cell subsets between liver transplant recipients with and without rejection

| Percentages (%) | Rejection (n=15) | Non-rejection (n=30) | P |
| --- | --- | --- | --- |
| ^a^IL-10^+^CD19^+^B cell | 1.59±1.48 | 1.42±1.05 | 0.662 |
| ^a^TGF-β^+^CD19^+^B cell | 1.68±1.26 | 1.36±1.02 | 0.373 |
| ^a^GrB^+^CD19^+^B cell | 3.06±1.68 | 2.91±1.61 | 0.760 |
| ^a^IL-10^+^CD19^+^B cell | 3.13±1.73 | 2.92±1.56 | 0.676 |
| ^a^TGF-β^+^CD19^+^B cell | 4.64±2.13 | 3.38±1.32 | 0.049 |
| ^a^GrB^+^CD19^+^B cell | 33.05±10.69 | 23.58±9.91 | 0.005 |

a, resting B cells; b, activated B cells

Table S4 Analysis of liver function and tacrolimus levels between liver transplant recipients with and without rejection

| Parameters | Rejection (n=15) | Non-rejection (n=30) | p |
| --- | --- | --- | --- |
| AST (U/L) | 164.07±99.75 | 57.27±32.68 | 0.001 |
| ALT (U/L) | 221.47±144.60 | 78.60±60.22 | 0.002 |
| TBIL (umol/L) | 53.15±50.35 | 28.89±16.31 | 0.088 |
| Tacrolimus levels (ng/ml) | 7.29±4.18 | 6.97±3.98 | 0.804 |

AST, aspartate transaminase; ALT, alanine amiotransferase; TBIL, total bilirubin

Table S5. Comparison of the Banff grades in the rejection group with the percentages of GrB^+^CD19^+^B cell and IFN-γ^+^CD4^+^T cell and the new marker

| Percentages (%) | Banff grade | | p |
| --- | --- | --- | --- |
|  | Mild (n=8) | Non-mild (n=7) |  |
| ^b^GrB^+^CD19^+^B cell | 28.96±7.68 | 37.74±12.25 | 0.115 |
| ^b^IFN-γ^+^CD4^+^T cell | 44.69±14.07 | 58.58±12.67 | 0.067 |
| New marker | 73.65±13.56 | 96.32±21.43 | 0.027 |

b, activated cells; GrB, granzyme B

Table S6 Comparison of the percentages of GrB^+^CD19^+^B cell and IFN-γ^+^CD4^+^T cell and the new marker in a validation cohort

| Percentages (%) | Rejection (n=23) | Non-rejection (n=78) | p |
| --- | --- | --- | --- |
| ^b^GrB^+^CD19^+^B cell | 34.46±11.26 | 25.99±10.89 | 0.002 |
| ^b^IFN-γ^+^CD4^+^T cell | 52.15±14.89 | 40.49±11.41 | 0.000 |
| New marker | 86.62±16.76 | 66.48±17.33 | 0.000 |

b, activated cells; GrB, granzyme B

Figure legends

**
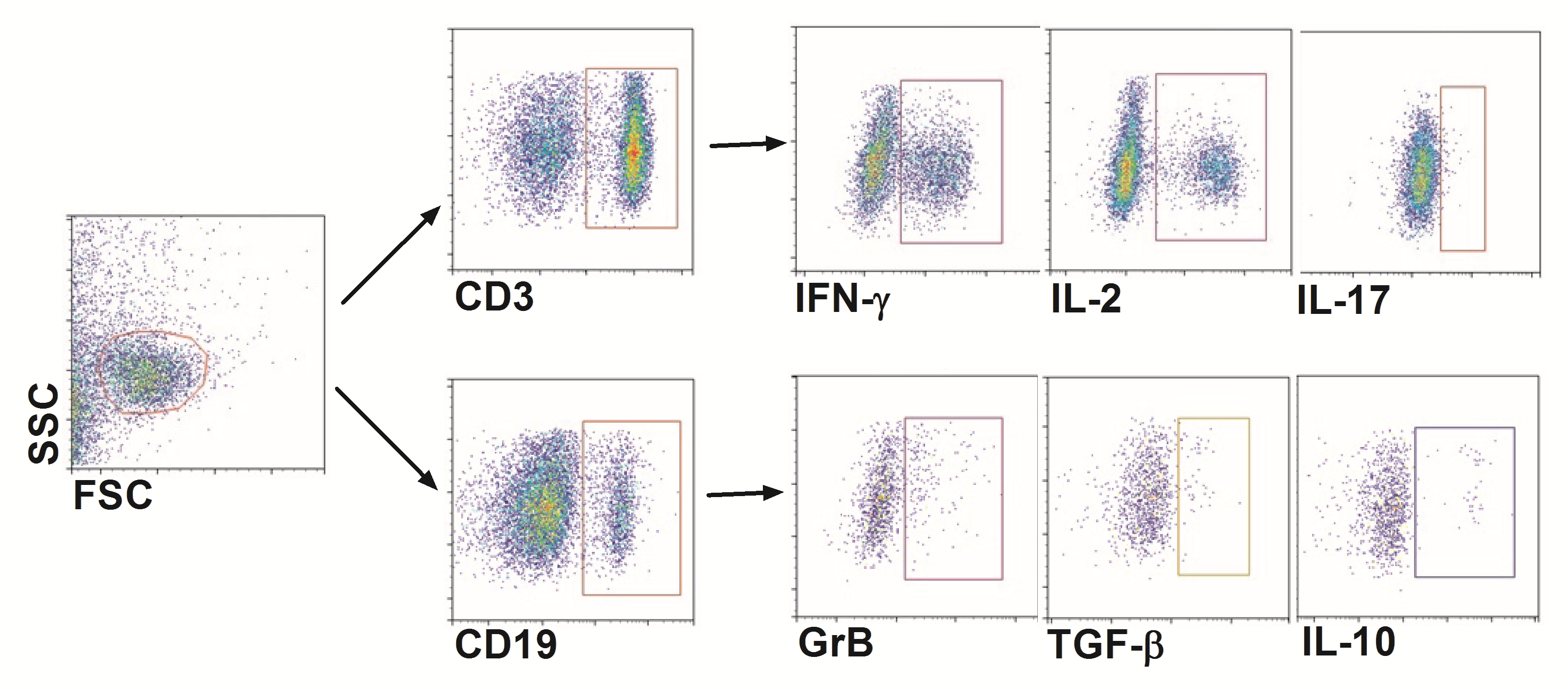
**

**Figure S1 Flow cytometry characterization of T cell subsets and B cell subsets**

**
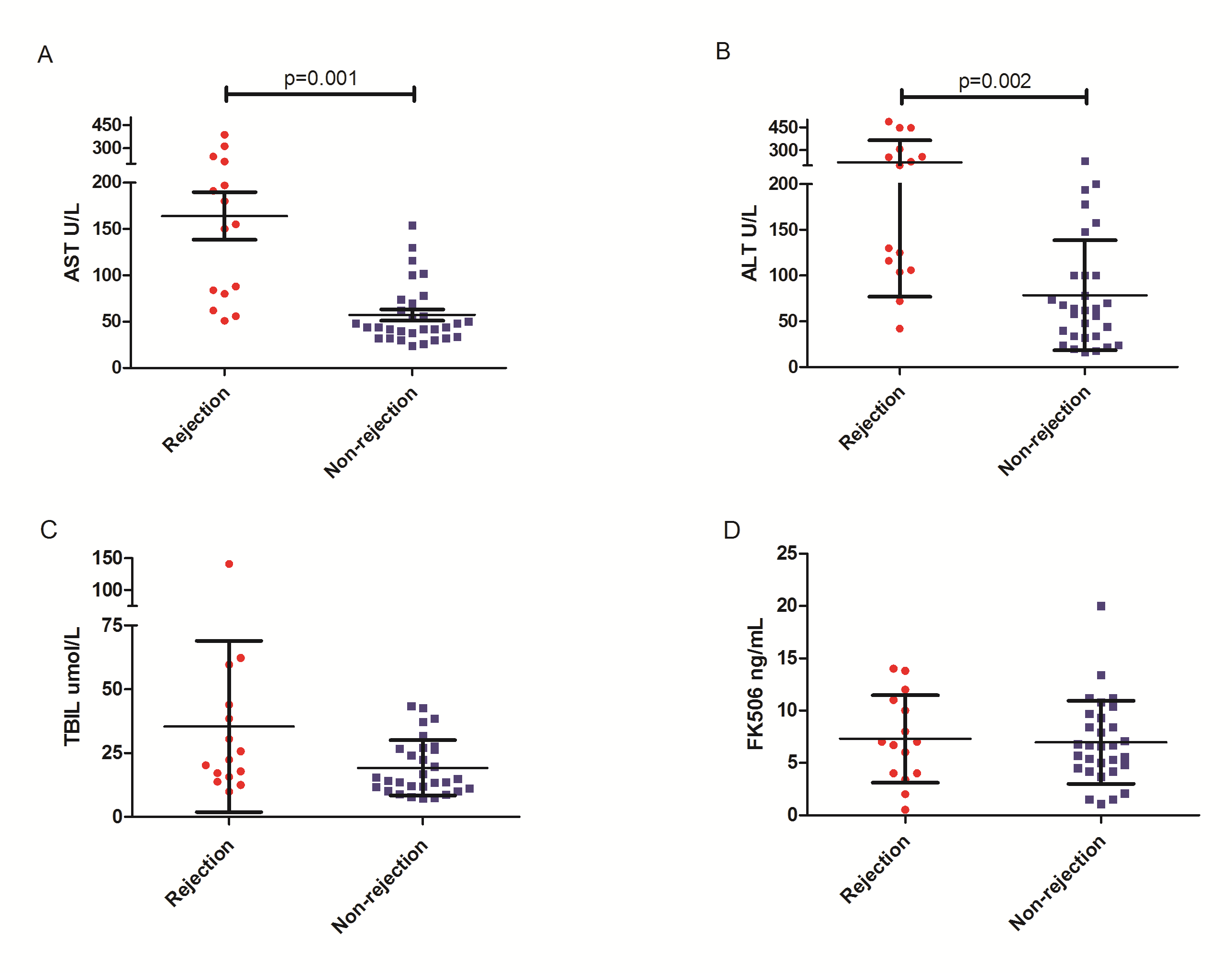
**

**Figure S2 AST and ALT deteriorate in liver transplant recipients with acute rejection.**

Comparison of levels of AST (A), ALT (B), TBIL (C) and FK506 (D) between liver transplant recipients with and without acute rejection. Bars represent mean and standard deviation. AST, aspartate transaminase; ALT, alanine amiotransferase; TBIL, total bilirubin
